# Primary Care Providers Perspectives of GLP-1 Receptor Agonists to Manage Recurrent Weight Gain after Metabolic Bariatric Surgery – a Qualitative Study

**DOI:** 10.1007/s11695-025-08408-0

**Published:** 2025-11-26

**Authors:** Liisa Tolvanen, Karin Mossberg, Lindsey M. Grace, Erin C. Standen, Sean M. Phelan, Daniel P. Andersson, Afton M. Koball

**Affiliations:** 1 Department of Medicine, H7, Karolinska Institutet, Stockholm, Sweden; 2 Department of Psychiatry and Psychology, Mayo Clinic, Rochester, USA; 3 General Practice / Family Medicine, School of Public Health and Community Medicine, Institute of Medicine, Sahlgrenska Academy, University of Gothenburg, Gothenburg, Sweden; 4 Research, Education, Development & Innovation, Primary Health Care, Region Västra Götaland, Gothenburg, Sweden; 5 Department of Family Medicine, Mayo Clinic, Rochester, USA; 6 Department of Psychological Sciences, Rice University, Houston, USA; 7 Division of Health Care Delivery Research, Robert D. and Patricia E. Kern Center for the Science of Health Care Delivery, Mayo Clinic, Rochester, USA; 8 Department of Endocrinology, C2:94, Karolinska University Hospital, Stockholm, Sweden

**Keywords:** Metabolic bariatric surgery, Primary care provider, GLP1-RA, Obesity management medications, Recurrent weight gain

## Abstract

**Introduction:**

Recurrent weight gain after metabolic bariatric surgery (MBS) affects 20–25% of patients, with research indicating that up to 87% of patients experience some post-MBS weight gain. Glucagon-like peptide-1 receptor agonists (GLP-1RAs) are increasingly being used to manage obesity in primary care. However, there remains a gap in understanding primary care providers (PCPs) perspectives on GLP-1RAs in the context of MBS.

**Methods:**

For this qualitative study, we recruited primary care providers (PCPs) (*n* = 38) from the United States and Sweden who had experience providing healthcare services in primary care after MBS. Individual semi-structured interviews were conducted between September 2024 and March 2025. We analyzed the transcribed interviews with reflexive thematic analysis.

**Results:**

The primary theme *“GLP-1RAs – Navigating Between Breakthrough and Uncertainty”* captures PCPs’ perception of the advent of GLP-1RAs as beneficial, while characterizing their uncertainty regarding GLP-1RA use in post-MBS care. PCPs welcomed the emergence of a new tool to address recurrent weight gain. However, patient requests for GLP-1RAs raised concerns about primary care prescription resources and access due to systematic barriers. PCPs identified a need for more knowledge and experience with GLP-1RA use post-MBS. They also recognized the changing role of MBS in treating obesity.

**Conclusion:**

PCPs perceived GLP-1RAs as a promising and effective tool for addressing recurrent weight gain after MBS, and that care options for patients had improved. Systematic barriers were perceived to have a negative impact on the effectiveness of treatment. More expert guidance and support to effectively treat patients with GLP-1RAs after MBS is needed.

## Highlights

PCPs regarded GLP-1RAs as promising therapies for managing recurrent weight gain.

GLP-1RAs were considered to change the role of MBS as a treatment for obesity.

The high-cost of GLP-1RAs was a perceived barrier for the long-term management.

More evidence and guidelines on interactions between GLP-1RA and MBS were requested.

## Introduction

Metabolic bariatric surgery (MBS) is the most effective and durable weight loss intervention currently available, facilitating sustained, optimal weight outcomes over the long-term [1–4]. Nevertheless, obesity is a chronic disease that may necessitate a combination of treatment modalities for long-term weight management [5]. Suboptimal clinical response, recurrent weight gain and the emergence of obesity-associated medical conditions may require additional interventions [6]. For example, some degree of recurrent weight gain affects up to 87% of individuals post-MBS, while 20–25% of patients may experience more significant recurrent weight gain [7] .

The introduction of new obesity medications, including Glucagon-Like Peptide-1 Receptor Agonist (GLP-1RA), such as semaglutide and dual-acting GLP-1RA/Glucose-Dependent Insulinotropic Polypeptide Receptor Agonist (GIP RA), such as tirzepatide, (hereafter referred to as GLP-1RAs except in participant quotes) have marked a significant advancement in the treatment of obesity [8, 9]. This is particularly relevant for patients experiencing suboptimal clinical response or recurrent weight gain following MBS, for whom additional treatment options have previously been limited [10–12]. Research on the efficacy of GLP-1RAs for addressing recurrent weight gain after MBS is emerging. Existing systematic reviews and meta-analyses suggest that the use of GLP-1RAs is both safe and effective for patients post-MBS, at least in the short term [11, 13–15]. Nonetheless, further research is necessary, as there are currently limited guidelines for the post-operative use of GLP-1RA [12].

Primary care providers (PCPs) are integral to the long-term support of patients after MBS, and therefore potentially suited to manage recurrent weight gain using GLP-1RAs [16]. Previous studies have highlighted both challenges (e.g., cost, side-effects) and opportunities (e.g., effective weight-loss, health improvements) in primary care [17, 18]. However, PCPs may lack knowledge on the use of GLP-1RAs after MBS to manage recurrent weight gain. Studying diverse healthcare systems and insurance frameworks, such as those in the United States and in Sweden, is crucial for enhancing our understanding of the challenges experienced in the real-world primary care settings with the effective management of recurrent weight after MBS. This international qualitative study aimed to understand PCPs’ perspectives on using GLP-1RAs in the management of recurrent weight gain following MBS and their perceptions of MBS in relation to GLP-1RA, to better recognize challenges, barriers, and future directions for optimized clinical obesity care.

## Materials and Methods

### Study Population

For this qualitative study, we employed purposive and snowball sampling methods [19] to recruit General Practitioners, Resident Physicians, Nurse Practitioners, Physician Assistants and District Nurses/Registered Nurses employed in primary care settings in the United States and Sweden between September 2024 and March 2025. We used our professional networks for recruitment, also the study participants could suggest new providers they knew could contribute with valuable information.

Eligible participants were those with experience in providing healthcare services in primary care to adult patients who had previously undergone MBS. A total of 56 PCPs were invited to participate in this study. Of these, twelve (*n* = 12) did not respond, six (*n* = 6) declined due to time constraints, limited interaction with post-MBS patients, or PCP perceived lack of representativeness of primary care. Among those who did not respond or declined were all PCPs represented, comprising eight (*n* = 8) men and ten (*n* = 10) women. PCPs received both oral and written information about the study and provided informed consent. Participation was voluntary, and no incentives were offered.

### Data Collection

We employed a qualitative design and conducted virtual interviews once with each PCP. Interviews were conducted in Sweden, using Swedish language, by the first author a registered dietitian (LT) and in the US, using English language, by the senior author, a clinical psychologist (AMK), both within specialty obesity care practices. We utilized a pilot-tested semi-structured interview guide that focused on post-MBS care in primary care and recurrent weight gain after MBS. The questions were designed to be open-ended (e.g., Tell me about your experience of treating patients who have experienced recurrent weight gain after MBS), allowing PCPs to express their perspectives and experiences freely. Follow-up probing questions were strategically employed to encourage PCPs to explore their reflections on the topic more deeply.

On average, the audio-recorded interviews lasted 39 min, with a range of 21–101 min. Transcriptions were performed simultaneously using an Artificial Intelligence (AI) generated function in Zoom© or Microsoft Teams©. The transcripts were carefully cross verified with the original audio to ensure accuracy. The validation process was conducted by the first and last author, who carefully manually reviewed the audio recordings with transcriptions multiple times to identify and correct any errors or inaccuracies introduced by the AI.

In this qualitative study, we aimed to collect rich and relevant data with various experiences. Therefore, the decision regarding the number of participants was guided by the depth and richness of the data needed to address our research aims, rather than by a predefined point of data saturation, consistent with Braun and Clarke’s Reflexive Thematic Analysis principles [20]. We kept the original language (Swedish/English) during the initial steps of the analysis to preserve the nuances of language. However, codes, themes and selected quotes were translated to English by bilingual Swedish researchers, and the English wording was verified by native English-speaking researchers in this project. All interviews were conducted confidentially, and all data files were de-identified to maintain participant confidentiality. If any participant gave especially unique information, the results were appropriately pseudonymized.

### Data Analysis

We manually analyzed the interview data transcripts with a step-by-step process utilizing inductive reflexive thematic analysis following the framework developed by Braun and Clarke [20]. The objective was to generate themes and subthemes that meaningfully captured the depth and complexity of PCPs’ perspectives. We specifically selected narratives from the collected data corpus that pertained to the experiences of primary care providers with GLP-1RAs in the context of recurrent weight gain following MBS. The data extracts were coded and systematically organized into sub-themes and one overarching theme, effectively encapsulating the essence of the participants’ narratives. The process of thematization involved iterative refinement and active collaboration within the research team.

### Reflexivity

Within an international and interdisciplinary team comprising experts in clinical obesity care, primary care, endocrinology, psychology, nutrition, epidemiology, public health, and weight stigma, we ensured reflexivity through regular meetings. Those meetings facilitated open discussions aimed to minimizing individual biases and preconceptions, particularly those of the first and last authors. The first (LT), second (KM), and last authors (AMK) engaged in collaborative dialogues to develop initial codes and themes, thereby enhancing reflexivity and deepening interpretative scientific rigor. All co-authors contributed to reviewing and refining the themes and sub-themes, offering critical insights that enriched the analytical process.

## Results

### Participants

In total, thirty-eight (*n* = 16 from the US and *n* = 22 from Sweden) PCPs were included as participants. Their experiences in following patients after MBS varied substantially, from meeting these patients only occasionally to providing routine follow-up visits annually. See Table 1 for participant characteristics. Not all demographic data was collected from the US providers, yielding missing demographic data, which is highlighted in Table 1.

**Table 1:** Participants’ characteristics (*N* = 38)

		USA (*n* = 16 (%))	Sweden (*n* = 22 (%))
Profession			
General Practitioner		8 (50.0)	11 (50.0)
Resident Physician		0 (0.0)	4 (18.2)
Physician Assistant		0 (0.0)	0 (0.0)
Nurse Practitioner^a^		8 (50.0)	0 (0.0)
District Nurse^b^, Specialist Nurse, Registered Nurse		0 (0.0)	7 (31.8)
Gender			
Female		14 (87.5)	18 (81.8)
Male		2 (12.5)	4 (18.2)
Age, years (range) (Swedish data)		-	46.1 (36–58)
Time since graduation (Swedish data)			
5–10 years		-	3 (13.6)
>10 years		-	19 (86.4)
Country of origin (Swedish data)			
Nordic		-	18 (81.8)
Outside of Europe		-	4 (18.2)

^a^ Nurse practitioner. In the United States, a Nurse Practitioner is an advanced practice registered nurse (APRN) with a total of six to 8 years of academic education. Their responsibilities include examining, diagnosing, and treating medical conditions, prescribing medications, and ordering diagnostic tests, paralleling the functions of a physician

^b^ District nurse. In Sweden, a district nurse is a registered nurse who has completed a specialist nursing program with total of at least 4.5 years of academic education. They primarily operate within primary health care. Their responsibilities often include working independently with prevention and the management of chronic conditions, can prescribe some medications such as vaccines

During the reflexive thematic analysis one primary theme “ GLP-1RAs: *Navigating between breakthrough and uncertainty”* and six sub-themes “*Bridging gaps in post-MBS care*,*” Tsunami of GLP-1RA requests*,*” “Systematic barriers to accessing GLP-1RA treatment*,*” “Limited knowledge and experience*,*” “Not a standalone solution”* and *“GLP-1RA transform the future for MBS”* were generated. Representative quotes are presented in Tables 2, 3, and 4 for each sub-theme.

### Primary Theme: GLP-1RAs: Navigating between Breakthrough and Uncertainty

The primary theme was a common thread throughout the data and captured the novel circumstances that the PCPs faced when considering use of GLP-1RAs in the treatment of obesity and in the context of recurrent weight gain after MBS. They expressed a positive attitude towards GLP-1RAs, noting their effectiveness and facilitation of discussions regarding weight with patients. However, there were concerns about whether primary care resources were adequate for handling prescription requests, and there was a perception that patients viewed GLP-1RAs as a miraculous solution which sometimes contrasted with PCPs’ clinical experiences. Additionally, PCPs sought guidelines on the proper use and safety of GLP-1RAs for patients after MBS. GLP-1RAs were considered to alter both patients’ treatment preferences and their own referral patterns to MBS.

### Sub-themes

***Bridging gaps in post-MBS care***, see Table 2 for representative quotes.

Primary care providers (PCPs) found it challenging to address recurrent weight gain after MBS when the only treatment tool available was individual-level, difficult to modify, health-related behavior change (e.g., dietary changes, physical activity). However, the advent of GLP-1RAs changed PCPs’ perceptions, offering a new, exciting, and potentially more effective treatment option, especially for patients who had undergone MBS several years ago with re-emerging comorbidities. PCPs described that GLP-1RAs offered renewed optimism for patients’ weight management. GLP-1RAs were considered as a tool for treating recurrent weight gain and as a complement to MBS for further weight reduction. GLP-1RAs were perceived as life-changing for patients which made prescribing them rewarding. Additionally, GLP-1RAs were perceived to enhance awareness and interest in obesity treatment. GLP-1RAs were suggested to be integrated as a routine component, as early as possible, in follow-up care after MBS, as PCPs had observed improvements in patients’ quality of life and health. Sometimes PCPs reported being unaware of patients’ previous MBS history due to the lack of documentation in patient records or that it was not disclosed during the consultations. PCPs reported increased confidence in addressing weight and obesity with patients due to GLP-1RAs, media coverage, and training availability. In addition, PCPs reported feeling more empowered in weight-related conversations when offering more than just advice on health-related behaviors.

***Tsunami of GLP-1RA requests***, see Table 2. for representative quotes.

PCPs in the US and in Sweden described experiencing an overwhelming demand for GLP-1RAs among patients with overweight and obesity, as well as patients after MBS. They perceived GLP-1RAs as *“trendy”* and noted that patients often saw them as a *“magical pill.”* They found that patients focused mainly on obtaining prescriptions rather than comprehensive obesity treatment. Generally, PCPs perceived their patients as well-informed and aware of specific GLP-1RAs they wanted. Media coverage was seen as driving increased demand. PCPs experienced that GLP-1RAs required significant resources for initiation and follow-up. They indicated that the deluge of requests was stressful and burdensome, as it resulted in a constant stream of patients needing support (e.g., with injection techniques, with various GLP-1RA dosages, pens, and syringes, and with managing side effects).

**Table 2:** Primary theme: GLP-1RAs - Navigating between breakthrough and Uncertainty. Representative quotes for Sub-themes “Bridging gaps in post-MBS care” and “Tsunami of GLP-1RA requests.” Note. […] refers to irrelevant content that has been removed. [Text] refers to descriptive content to enhance comprehension

Sub-theme: Bridging gaps in post-MBS care
General Practitioner, Sweden: *“It is so difficult with lifestyle advice* [for patients with recurrent weight gain], *and what good does it do? How much resources should be spent on something that might not change your mind?”* (Participant #30) General Practitioner, US: *“*[Referring to a patient who experienced recurrent weight gain after MBS] *The GLP-1 has worked well for* [the patient] *and* [the patient] *said*,* it’s the thing that’s worked the best since* [patient’s] *bariatric surgery.”* (Participant #3) Nurse practitioner, US: *“*[A patient with recurrent weight gain] *is still losing weight* [by using GLP-1RAs] *and feels great and is doing things that wasn’t possible because of weight. In fact*,* we’re decreasing* […] *blood pressure medication. So*, [patient] *has that renewed sense of hope because* […] *lost weight.”* (Participant #8). District Nurse, Sweden: “*With these medications* [GLP-1RAs], *there is an extra chance* [to lose weight] *for them* [patients with recurrent weight gain].*”* (Participant #24). Nurse Practitioner, US: *“It’s a very rewarding to do* [prescribe GLP-1RAs] *in primary care*,* probably and in endo* [Endocrine clinics] *because patients are seeing results*,* and it can be life changing.”* (Participant #13) General Practitioner, Sweden: *“Today*,* we have drugs* [GLP-1RAs], […]. *You must start thinking differently. This is because there are more opportunities to provide support if you regain weight.”* (Participant #38) General Practitioner, Sweden: “[Patient with recurrent weight gain] *ended up being prescribed medication* [GPL-1RA]. […] *also managed to lose weight and has since undergone hip surgery.”* (Participant #36). District Nurse, Sweden: *“It’s a bit frightening*,* because sometimes important things might be overlooked*,* maybe no one* [patient] *mentioned*,* or the physician didn’t notice that this patient had surgery* [MBS] *ten to fifteen years ago. If that is missed*,* it could lead to entirely wrong conclusions.”* (Participant #24) Nurse Practitioner, US: *“I think there’s enough out there in terms of some of these weight loss options. I have more patients actually coming to me asking me about them versus me even having to bring it up. So that makes it kind of a little bit easier.”* (Participant #9)
Sub-theme: Tsunami of GLP-1RA requests
General Practitioner, Sweden: *“They are often young and healthy or have only seen me occasionally for minor issues like a cold. Now*,* however*,* they’re reaching out solely to request a prescription*,* nothing more. They are not looking for advice or any other form of care.”* (Participant #27). General Practitioner, US: *“Now with the GLP-1 agonist information and the patient’s knowledge of it is everywhere*,* you know*,* everyone is informed.”* (Participant #10). General Practitioner, Sweden: “*A lot has happened with the new drugs* [GLP-1RA] *in the past year. And there has been a tsunami of patients contacting primary care.” (*Participant #27) General Practitioner, US: *“Everybody wants the GLP1s”* (Participant #7) General Practitioner, US: *“It* [GLP-1RA] *is also trendy.”* (Participant #6) District Nurse, Sweden: “*It’s like an explosion. I have at least four patients this week who are being put on* [name of GLP-1RA] *or* [name of GLP-1RA] *and are going to have introductions and follow-ups. I am going to give them everything they need*,* so they know what they are getting into*,* and receive the right help.”* (Participant #24) Resident Physician, Sweden: *“Several patients per week who are bothered by their excess weight want to talk and get help* [GLP-1RA]. *Many perceive it as […] that I just prescribe and that’s it.”* (Participant #20) General Practitioner, Sweden: *“Patients read the newspapers and follow along and demand GLP analogs*,* of course. Because they have heard that they* [GLP-1RAs] *are effective*.” (Participant #35)

***Systematic barriers to accessing GLP-1RA treatment***, see Table 3 for representative quotes.

PCPs experienced multiple obstacles preventing patients from initiating or maintaining GLP-1RA treatment. Their main concern was surrounding the financial burden for patients, particularly a lack of insurance coverage or financial subsidies, negatively affecting care continuity. PCPs noted that patients’ financial resources directly influenced their access to treatment. In Sweden, patients must bear full costs of medication, with obtaining national insurance support deemed nearly impossible. In the US, PCPs expressed concerns about inequitable access to the medications arising from lack of insurance coverage or coverage limits. PCPs expressed interest in subsidies for GLP-1RA treatment, however, they also worried about the societal financial burden of universal coverage. Patients often found insurance issues frustrating, especially when reaching benefit limits according to PCPs.

Although the availability of GLP-1RAs had increased over the past year, PCPs also expressed concerns about access to GLP-1RAs for patients who have conditions for which the medications are covered or subsidized (e.g., those with type 2 diabetes), while remaining optimistic that availability and prices would improve over time. In addition, PCPs, especially those in Sweden, felt that the lack of guidelines for the use of GLP-1RAs in post-MBS care to be problematic, and that they were sometimes limited in offering these new treatments due to regional restrictions. They expressed concern that their patients could obtain prescriptions for GLP-1RAs through various physician-led digital platforms (mobile applications or web-based services). This could result in PCPs being unaware of their patients’ GLP-1RA treatment, thereby creating uncertainty regarding follow-up, patient safety, and increasing patients’ out-of-pocket costs. They believed that this approach was not beneficial to the patients or society.

***Limited knowledge and experience***, see Table 3 for representative quotes.

PCPs reported variability in prescribing and managing GLP-1RAs after MBS care. While some felt confident, others lacked experience. They perceived a need for more research and clinical experience and expressed interest in using GLP-1RAs in primary care, while noting variability in clinical practices. Given these therapies’ novelty, particularly post-MBS, clear guidelines were needed. PCPs’ GLP-1RA prescribing practices for post-MBS patients aligned with their approach to treating patients without previous MBS. PCPs’ considerations focused on long-term efficacy and safety, particularly regarding nutrition and body composition.

In addition, PCPs expressed concerns about patients struggling with appetite regulation despite GLP-1RA treatment. Management of side effects and nutritional guidance were highlighted as risks to patients after MBS to consider. PCPs found it challenging when patients experienced recurrent weight gain despite GLP-1RA use or when treatment was ineffective. Recurrent weight gain after discontinuing GLP-1RAs and ongoing eating difficulties were also described. The PCPs noted that body image and mental health issues often persisted during treatment.

**Table 3:** Primary theme: GLP-1RAs - Navigating between breakthrough and Uncertainty. Representative quotes on sub-themes “Systematic barriers to accessing GLP-1RA treatment” and “Limited knowledge and experience”. Note. […] refers to irrelevant content that has been removed. [Text] refers to descriptive content to enhance comprehension

Sub-theme: Systematic barriers to accessing GLP-1RA treatment.
General Practitioner, US: *“I have philosophical and ethical quandaries about recommending a 300-400 a month medicine for you take for the rest of your life with no *[financial] *relief in sight.”* (Participant #6) District Nurse, Sweden: *We have tried to apply for Additional Cost Allowance* [reimbursement from the Swedish Social Insurance Agency] *for some patients. However*,* we have been rejected*,* and it has really been patients for whom we have assessed that there is a need.”* (Participant #33). General Practitioner, Sweden: “*I am pleased that there are effective medicines* [GLP-1RAs], *in case they* [patients] *can afford them.” (Participant #18).* Nurse Practitioner, US: “*Almost all my patients are on* [name of the insurance] *insurance*,* and all of them are coming up on this cap of funds* [to cover weight loss medications]. *And so*,* I’m now in this new zone of `Oh*,* my gosh! What are we gonna do? ` I don’t know that if you ask me a year from now*,* if I would feel like these were such a magic pill. I’m gonna have a lot of unhappy people soon* [they’re maxed out on their insurance].*”* (Participant #13) General Practitioner, Sweden: *“Now*,* it’s kind of generalized that they* [patients] *call in* [to an app-based doctor] *saying*,* ‘But my BMI is like this*,* and I feel like this‘*,* ‘OK*,* try* [name of a GLP-1RA medicine]‘ *then it costs us in society more than if we structure it* [GLP-1RA prescriptions].” (Participant #29) General Practitioners, Sweden: *“Take National guidelines*,* when they state that this* [MBS] *should be prioritized over GLP-1 analogs*,* it often comes down to an expert group*,* maybe just three people*,* deciding that ‘this is what we should prioritized’.”* (Participant #30) District Nurse, Sweden; “[It’s difficult to help patients] *because* [GLP-1RAs] *are not included in the guidelines.”* (Participant #19) General Practitioner, Sweden: “*You go in and check the list of medications. There is a national drug list*, […] *and then you see*,* yes*,* they are on it* [GLP-1RA] *and then they haven’t mentioned it. There are a lot of them*.” (Participant #32)
Sub-theme: Limited knowledge and experience
General Practitioner, US: *“GLP-1s are a mixed blessing. I have been in this game a long time*,* so I am interested to see long term what we see.”* (Participant #10) General Practitioner, Sweden: “[If a patient with recurrent weight gain seeks primary care]. *Currently*,* there are other weight-reducing drugs that can be used*,* such as GLP-1 analogs. However*,* we do not prescribe them. We have not learned how to use them. These are not included in the* [healthcare] *coverage.* […] *I have not prescribed them regularly or I do not initiate that type of treatment myself.”* (Participant #26) Nurse Practitioner, US: *“The other thought I have had with* [GLP-1RA] *medication options as a primary care provider*,* and I don’t know what the answer is. Are these patients who have had gastric bypass*,* is putting them on weight loss drugs* [GLP-1RA] *the right thing or should they better qualify for revision surgery but there’s not a lot of resources around those questions for primary care providers? So*,* what is best practice? What is best care for those patients?”* (Participant #8) General Practitioner, Sweden: “*There have been some* [patients] *who have asked for those drugs* [GLP-1RAs], *and then I have felt a little unsure about the effect of those who have had surgery* [MBS]. *I have probably asked the obesity clinic* [specialist] *what they think is reasonable in this situation. I suppose that they have answered that it is completely okay to treat even those who have undergone surgery* [MBS] *but regained weight.”* (Participant #35) District Nurse, Sweden: “*I think it is difficult with medication* [GLP-1RA]. *How long* [time] *should they* [the patients] *take them?”* (Participant #33) Nurse Practitioner, US:” *If it’s the second thing* [MBS first], *they already did the gastric surgery* [MBS], *regained the weight. Now they want to try this* [GLP-1RA], *you have a little bit more hesitation of*,* is this really gonna work or not?*” (Participant #11)

***Not a standalone solution***, see Table 4 for representative quotes.

PCPs emphasized that GLP-1RAs after MBS should be integrated with behavioral interventions and medical nutrition therapy for sustainable outcomes, stressing the need for support in health-related behavior changes for optimal long-term results. Some referred patients to dietitians and encouraged physical activity, emphasizing individualized care to sustain patient motivation. However, comprehensive support was not always feasible in all settings, and they found it challenging to advise additional treatment and meet patient expectations if they only demanded GLP-1RA prescriptions. The PCPs expressed that they were convinced that additional support could help patients set realistic goals, improve satisfaction, and enhance adherence.

***GLP-1RA transform the future for MBS***, see Table 4 for representative quotes.

The introduction of GLP-1RAs had influenced how PCPs viewed MBS. They observed that patients in both the US and Sweden were now more likely to explore GLP-1RAs instead of seeking referrals for MBS or considering MBS options abroad. However, PCPs reported that patients did not always seem interested either in MBS or GLP-1RAs, asking PCPs to adjust their approach to *“meet patients there where they are.”*

The PCPs perceived the future of MBS in the treatment of obesity as uncertain with the emergence and efficacy of GLP-1RAs. They reported a shift in their attitudes towards referring patients for MBS; preferring to initiate GLP-1RAs before considering referrals, whereas others expressed reservations about making such referrals. PCPs emphasized that some patients would need MBS based on medical history and health goals. For instance, those who cannot afford GLP-1RAs, do not respond to treatments, or have body mass index over 45–50 kg/m^2^ could be recommended MBS. PCPs also suggested MBS could be more cost-effective long-term compared to continuous GLP-1RA use. Additionally, GLP-1RAs were viewed as a potential step towards surgery, particularly for patients who achieve weight loss with medication and want to maintain these improvements.

**Table 4:** Primary theme: GLP-1RAs - Navigating between breakthrough and Uncertainty. Representative quotes on sub-themes “Not a standalone solution” and “GLP-1RA transform the future for MBS”. Note. […] refers to irrelevant content that has been removed. [Text] refers to descriptive content to enhance comprehension

Sub-theme: Not a standalone solution
General Practitioner, US: “*Lifestyle is the backbone.”* (Participant #6) General Practitioner, Sweden: *“There is no miracle* [in obesity treatment] *whether it is medication* [GLP-1RA] *or* [other] *treatment that you receive in one visit.”* (Participant #38*)* General Practitioner, US: *“I think we’ve been kind of coursed a little bit into prescribing GLP-1 agonists by patients without ‘Oh*,* no*,* I don’t have time to see the dietitian. I know what I’m supposed to do.’ So*,* I think we’re prescribing*,* and myself included*,* without that key lifestyle/education piece. And I think that we do our patients a disservice. But it’s that balance of meeting the patient’s expectation. And you know what’s the correct thing to be doing.”* (Participant #10) Nurse Practitioner, US: “*I think the important thing for me is having that accountability discussion again. Surgery* [MBS] *is only going to get you so far*,* medications* [GLP-1RAs] *are only going to get you so far*,* you have to be accountable for the things that you can modify.”* (Participant #9) District Nurse, Sweden: *“Patients may have stomach problems*,* reflux*,* or other conditions*,* and these medications* [GLP-1RAs] *are completely wrong. Therefore*,* the patient should not initiate treatment.* [The physicians need] *to ask how the patient feels* [on GLP-1RAs].” (Participant #23)
Sub-theme: GLP-1RA transform the future for MBS
General Practitioner, US: *“I think the interesting thing is that with the advent of weight loss medications*,* the surgery* [MBS] *has now*,* just in the last year seemed to be a little less prominent and that the GLP-1 agonists have been taking over.”* (Participant #16) General Practitioner, US: *“I’m certainly referring fewer people* [to MBS] *now that we have GLP1-RAs”.* (Participant #6). General Practitioner, Sweden:*” I think it* [MBS] *is a very bad option. I say that ‘I do not know anyone*,* that I have referred*,* who has maintained their low weight* [after MBS], *and that there are risks of surgery and side effects ‘. My current expectations are just low*,* meaning that I no longer send* [a referral] *for this* [MBS]. *That is* [what I inform], *when they* [patients] *call me and want to be referred to* [MBS]. *Yes*,* I am quite negative about surgery.”* (Participant #36). General Practitioner, US: *“With the GLP1s*,* I think that you could have a* […] *better success* [than with MBS].” (Participant #1). General Practitioner, US: *“One patient comes to mind who said prior to a GLP-1 that* [patient] *would have never considered MBS. But now* [patient] *is* [considering it] *because* [patient] *feels so much better* [after taking GLP-1RA and losing weight], *and* [patient] *doesn’t want to go back.”* (Participant #3) General Practitioner, Sweden: “*Those who can afford to pay* [GLP-1RA] *will get that treatment then. Those who cannot afford to pay* [GLP-1RA] …*I think*,* it might be primarily those who cannot afford to pay who will undergo surgery* [MBS].*”* (Participant #27)

## Discussion

Results from this international qualitative study indicate that PCPs have experienced a change in how obesity can be treated, including for those with recurrent weight gain following MBS with the advent of GLP-1RAs. PCPs were enthusiastic about the potential of GLP-1RAs as breakthroughs in treatment. However, they were facing knowledge gaps, systemic hurdles, and uncertainties regarding the integration of these medications with surgical interventions. This situation forced PCPs to navigate between medical breakthrough and the uncertainty in the healthcare system. PCPs regarded GLP-1RAs as “game changer” in medical science similarly to the study of Holtrop et al. [18], recognizing their potential as a new “lifeline” and an effective “tool” for patients who had struggled with recurrent weight gain after MBS. On the other hand, providers also described uncertainties and concerns about how and when to use GLP-1RA after MBS without evidence-based guidelines. This aligns with previous findings indicating that General Practitioners and Physicians are not adequately familiar with prescribing GLP-1RAs to their patients [21, 22]. Consequently, patients may encounter difficulties in obtaining effective treatment. In our study, PCPs identified obstacles to treatment that aligned with those reported in previous research including systematic barriers with financial subsidies and lack of insurance [17, 18, 21, 22]. These challenges could cause ethical stress in PCPs when knowing that effective treatment exists, but patients cannot access the treatment due to financial concerns.

PCPs described the current time as a historical period when GLP-1RAs have transformed obesity care in the US and Sweden. Like the work of Andreassen et al., PCPs in the present study had experienced a “wave” of patients demanding prescriptions of GLP-1RAs in primary care [17]. Despite the substantial differences in healthcare systems and insurance structures between the US and Sweden, the barriers to effective and sustainable obesity care following MBS were found to be surprisingly similar. In both, the US and Sweden, PCPs expressed challenges with patient access to medications, primarily due to financial hurdles. In the US, PCPs discussed insurance barriers and the high cost of medications. In Sweden, PCPs similarly described the high cost of medications, typically necessitating that patients pay out-of-pocket to access GLP-1RAs. Despite the “universal” tax-funded Swedish health and medical care system where most medications are included [23], PCPs across countries described limited government resources to support financial feasibility of accessing these medications. The findings indicate that the current treatment landscape poses significant challenges for primary care regardless of healthcare settings, both in terms of resources and economic considerations.

The lack of reimbursement for GLP-1RA treatments have been highlighted as a factor that could either improve or worsen experiences related to weight stigma [24]. Individuals who can afford these medications and therefore lose weight may experience reduced levels of stigma, whereas those who cannot afford them may continue to encounter high levels of weight-related blame and shame. However, the relationship between financial resources for GLP-1RAs and weight stigma is complicated. Emerging evidence suggests that people may also face stigma for using GLP-1RA medications, as some view the medications as “taking the easy way out,” particularly as compared to using behavioral weight loss approaches [25]. Stigma for any of these reasons (e.g., due to one’s weight, due to using GLP-1RA medication, due to not being able to afford GLP-1RA medication) is likely to worsen existing socioeconomic disparities in obesity care and lead to harmful health consequences [26]. Thus, will be important to employ multi-pronged approaches to ensure high-quality care for patients post-MBS. PCPs must work to eliminate both weight stigma and GLP-1RA-related stigma, and healthcare policymakers must support PCPs in their efforts to provide effective care by advocating for equitable allocation of resources. However, these decision-makers seem to frequently fall short in effectively addressing the challenges that accompany these responsibilities especially when it comes to obesity care [27].

One issue that PCPs desired guidance on was whether conversations about GLP-1RAs increase or decrease patients’ adherence to other forms of obesity treatment (i.e., behavioral, surgical interventions). PCPs perceived that patients’ increased interest in GLP-1RAs can make them reluctant to integrate additional treatment options to support their health-related behaviors into their treatment plan. On the other hand, PCPs perceived that GLP-1RAs serve as a “bridge to MBS,” therefore increasing interest in such treatment, an idea echoed in the recent International Federation for the Surgery and Other Therapies for Obesity (IFSO) position statement [28]. PCPs also indicated that either patients or the referring parties (themselves) might lose interest in MBS due to the enhanced effectiveness of GLP-1RAs, which, unlike MBS, are not permanent. Moreover, some PCPs perceived GLP-1RA and MBS as equivocal despite existing evidence showing superiority of MBS due to the sustained weight loss outcomes and reduced cardiovascular risk and mortality [29, 30]. This misconception underscores the need for better dissemination of long-term outcome data to primary care as MBS offers unmatched long-term weight loss and mortality benefits in eligible patients, which some PCPs in our study were not fully aware of.

### Limitations and Strengths

While previous work has focused on PCP perspectives on weight-loss medications broadly [17, 18], the current study zeroes in on provider perspectives specifically after MBS, an important contribution to the literature. Given that GLP-1RAs represent a novel treatment option for recurrent weight gain after MBS on a global scale, the perceptions and experiences vary among PCPs. However, the main strength of this qualitative study lies in its international scope, which enhances the transferability to different contexts. Overall, PCPs were excited about GLP-1RAs’ potential (breakthrough) but were grappling with knowledge gaps, system hurdles, underlying stigma, and questions about how these drugs fit with surgery (uncertainty).

Despite the strengths of the current international study with a large sample size, some limitations must be acknowledged. Qualitative research prioritizes depth over breadth; therefore, while the findings offer valuable insights, they may not reflect all PCPs. Our inclusion criteria required participants to have some experience with MBS, ensuring perspectives grounded in relevant clinical experience but excluding PCPs with no exposure to this patient population. Nonetheless, participants represented a range of familiarity with MBS and GLP-1RA prescribing, reflecting the variability typical of “real-world” primary care settings.

We actively strived for a variation of perspectives and experiences in our study. However, a limitation is that the most participants were women. Future studies could specifically focus on to explore male PCPs perspective. It is a strength for our results that we could include healthcare professionals with different experiences. District Nurses and Registered Nurses were more actively engaged in supporting patients with changes of health-related behaviors, in contrary General Practitioners and Nurse Practitioners had more of a role of managing patients’ treatment and prescribing GLP-1RAs.

This study captured PCPs attitudes at one time point. However, given the evolving landscape of GLP-1RAs, these perspectives may change as new guidelines emerge, prevalence increases, or access, coverage, and cost evolve. Recruitment used purposive and snowball sampling, appropriate for hard-to-reach populations, but potentially introducing selection bias. Participants who volunteered may not represent the broader PCP population, as those with specific interests may have been more willing to participate in the study. This limitation affects the interpretation of the results. The participants showed diverse experiences and perspectives. Despite interviewers’ efforts to encourage openness in participant responses, some viewpoints may be underrepresented, especially given the novelty surrounding GLP-1RAs. Furthermore, PCPs may have provided socially acceptable answers rather than their true experiences due to social desirability bias.

The interviewers aimed to collect rich, comprehensive, and in-depth data, however the duration of the interviews varied. While the length of an interview does not inherently determine its quality, shorter interviews may lack completeness, and longer interviews may not necessarily guarantee the desired depth [19]. To mitigate potential bias during data collection, we employed only two interviewers, one in each country, to ensure consistent interview quality irrespective of time constraints. Researcher reflexivity was maintained through interdisciplinary discussions throughout the research process. Our professional background as obesity researchers and clinicians may have both limited and strengthened our interpretation of interview data. The reflexivity efforts aimed to increase awareness of this influence.

Although including participants from both Sweden and the United States strengthens the study by capturing perspectives across two distinct healthcare systems, the applicability of these findings to other contexts may be limited. Differences in healthcare organization, cultural attitudes toward obesity, and access to treatment may influence the manifestation of these themes in other regions. Future research, including providers from additional countries and settings, particularly low- and middle-income contexts, would help clarify the global relevance of these findings.

### Practical Implications and Future Directions

By understanding PCPs perspectives and generating themes presented here, several recommendations for action reveal. Our findings suggest an urgent need for official guidelines on post-MBS use of GLP-1RAs with PCPs included in developing these given their frequent role in providing long-term post-operative care. As a start, international guidelines are emerging e.g., IFSO recommends considering GLP-1RAs before revision MBS in cases of recurrent weight gain [10]. However, PCPs in our study were largely unaware of such guidance, indicating a gap between specialist consensus and primary care practice.

Clear guidelines and recommendations on when to use GLP-1RAs before and after MBS would be helpful for PCPs to do shared-decision making with their patients and to provide the most safe and effective treatment approaches to treat recurrent weight gain. PCPs expressed a lack of clarity about use of GLP-1RAs after MBS, and this was complicated by not always knowing a patient’s full medical history including whether they had even had MBS in the past. Concerns regarding nutritional challenges, effects on body composition, and MBS-related side effects were emphasized by PCPs. These aspects are also highlighted by IFSO in a Delphi Consensus Position Statement, advising GLP-1RA treatment before revisional surgery [10].

The identified themes brought up a few additional considerations that warrant further research. For instance, PCPs commented on patients wanting medications as a “quick fix,” which may reflect underlying weight bias, as it is consistent with findings that GLP-1RA users are stigmatized for “taking the easy way out” [25]. However, they also noted that bringing up GLP-1RAs has made it easier to have weight-related conversations with patients. Thus, it is not immediately clear whether PCPs having more conversations about GLP-1RAs would increase or decrease patients’ experiences of stigma. On the one hand, having additional treatment options for post-MBS patients experiencing recurrent weight gain may help PCPs have more positive, supportive conversations with their patients, but on the other hand, if PCPs continue to view patients seeking GLP-1RAs as wanting “a magical pill,” those conversations may still be laden with stigma. Future research should examine the role that GLP-1RAs play in weight-related communication and stigma for patients post-MBS. At the systems level, it will be important to examine how lack of insurance coverage and the exorbitant cost of GLP-1RA medications makes obtaining this treatment so difficult for patients, particularly because those financial barriers may affect patients’ choice to use GLP-1RAs pre- or post-MBS [24].

## Conclusion

PCPs perceive GLP-1RA therapies as a promising treatment option for recurrent weight gain post-operatively yet acknowledge that it is a new landscape with more research and guidance needed. They believed that the role of MBS has already changed as a result of GLP-1RAs and may further change over time with GLP-1RAs becoming more effective, prevalent, affordable, and available for patients.

## Data Availability

Data availitibility is not possible due human research participants may present a risk of reidentification if shared openly.

## References

[CR1] 1. Schauer PR, Mingrone G, Ikramuddin S, Wolfe B. Clinical outcomes of metabolic surgery: efficacy of glycemic control, weight loss, and remission of diabetes. Diabetes Care. 2016;39(6):902–911. 10.2337/dc16-0382. 10.2337/dc16-0382 PMC5864131 27222548

[CR2] 2. Aminian A, Zajichek A, Arterburn DE, Wolski KE, Brethauer SA, Schauer PR, et al. Association of metabolic surgery with major adverse cardiovascular outcomes in patients with type 2 diabetes and obesity. JAMA. 2019;322(13):1271–1282. 10.1001/jama.2019.14231. 10.1001/jama.2019.14231 PMC6724187 31475297

[CR3] 3. Karlsson J, Taft C, Rydén A, Sjöström L, Sullivan M. Ten-year trends in health-related quality of life after surgical and conventional treatment for severe obesity: the SOS intervention study. Int J Obes (Lond). 2007;31(8):1248–1261. 10.1038/sj.ijo.0803573. 10.1038/sj.ijo.0803573 17356530

[CR4] 4. Eisenberg D, Shikora SA, Aarts E, Aminian A, Angrisani L, Cohen RV, et al. 2022 American Society of Metabolic and Bariatric Surgery (ASMBS) and International Federation for the Surgery of Obesity and Metabolic Disorders (IFSO) indications for metabolic and bariatric surgery. Obes Surg. 2023;33(1):3–14. 10.1007/s11695-022-06332-1. 10.1007/s11695-022-06332-1 PMC9834364 36336720

[CR5] 5. Busetto L, Dicker D, Frühbeck G, Halford JCG, Sbraccia P, Yumuk V, et al. A new framework for the diagnosis, staging and management of obesity in adults. Nat Med. 2024;30(9):2395–2399. 10.1038/s41591-024-03095-3. 10.1038/s41591-024-03095-3 38969880

[CR6] 6. Haddad A, Suter M, Greve JW, Shikora S, Prager G, Dayyeh BA, et al. Therapeutic options for recurrence of weight and obesity related complications after metabolic and bariatric surgery: an IFSO position statement. Obes Surg. 2024;34(11):3944–3962. 10.1007/s11695-024-07489-7. 10.1007/s11695-024-07489-7 39400870

[CR7] 7. Voorwinde V, Steenhuis IHM, Janssen IMC, Monpellier VM, van Stralen MM. Definitions of long-term weight regain and their associations with clinical outcomes. Obes Surg. 2020;30(2):527–536. 10.1007/s11695-019-04210-x. 10.1007/s11695-019-04210-x 31677016

[CR8] 8. Wilding JPH, Batterham RL, Calanna S, Davies M, Van Gaal LF, Lingvay I, et al. Once-weekly semaglutide in adults with overweight or obesity. N Engl J Med. 2021;384(11):989–1002. 10.1056/NEJMoa2032183. 10.1056/NEJMoa2032183 33567185

[CR9] 9. Jastreboff AM, Aronne LJ, Ahmad NN, Wharton S, Connery L, Alves B, et al. Tirzepatide once weekly for the treatment of obesity. N Engl J Med. 2022;387(3):205–216. 10.1056/NEJMoa2206038. 10.1056/NEJMoa2206038 35658024

[CR10] 10. Cohen RV, Busetto L, Levinson R, Le Roux CW, Salminen P, Prager G. International consensus position statement on the role of obesity management medications in the context of metabolic bariatric surgery: expert guideline by the International Federation for the Surgery of Obesity and Metabolic Disorders (IFSO). Br J Surg. 2024;111(12):znae283. 10.1093/bjs/znae283 10.1093/bjs/znae283 39612579

[CR11] 11. Kellett J, Soliman SS, Podwojniak A, Minkanic M, Kumar G, Goodwin B, et al. The efficacy of glucagon-like peptide-1 (GLP-1) receptor agonists for insufficient weight loss or regain after metabolic/bariatric surgery: a systematic review and meta-analysis. Obes Surg. 2025;35(3):1127–1134. 10.1007/s11695-025-07723-w 10.1007/s11695-025-07723-w 39910018

[CR12] 12. Cohen RV, Park JY, Prager G, Bueter M, le Roux CW, Parmar C, et al. Role of obesity-management medications before and after metabolic bariatric surgery: a systematic review. Br J Surg. 2024;111(12):znae284. 10.1093/bjs/znae284 10.1093/bjs/znae284 39612581

[CR13] 13. Esparham A, Mehri A, Dalili A, Richards J, Khorgami Z. Safety and efficacy of glucagon-like peptide-1 (GLP-1) receptor agonists in patients with weight regain or insufficient weight loss after metabolic bariatric surgery: a systematic review and meta-analysis. Obes Rev. 2024;25(11):e13811. 10.1111/obr.13811 10.1111/obr.13811 39134066

[CR14] 14. Nie Y, Zhang Y, Liu B, Meng H. Glucagon-like peptide-1 receptor agonists for the treatment of suboptimal initial clinical response and weight gain recurrence after bariatric surgery: a systematic review and meta-analysis. Obes Surg. 2025;35(3):808–822. 10.1007/s11695-025-07733-8. 10.1007/s11695-025-07733-8 39948306

[CR15] 15. Jamialahamdi T, Mirhadi E, Almahmeed W, Virani S, Eid AH, Al-Rasadi K, et al. Impact of semaglutide administration on weight loss after bariatric surgery: a meta-analysis. Obes Surg. 2025;35(7):2490–2497. 10.1007/s11695-025-07848-y. 10.1007/s11695-025-07848-y 40418526

[CR16] 16. Tolvanen L, Mossberg K, Standen EC, Grace LM, Lebow JR, Phelan SM, et al. Integrative strategies in primary care: addressing recurrent weight gain post-metabolic and bariatric surgery. Obes Sci Pract. 2025;11(4):e70087. 10.1002/osp4.70087. 10.1002/osp4.70087 PMC12336673 40791996

[CR17] 17. Andreassen P, Jensen SD, Bruun JM, Sandbæk A. Managing the new wave of weight loss medication in general practice: a qualitative study. Clin Obes. 2024;14(3):e12666. 10.1111/cob.12666. 10.1111/cob.12666 38660941

[CR18] 18. Holtrop JS, Tietbohl C, Perreault L, Connelly L, Smith PC, Williams J. Primary care patient and practice member perspectives on weight loss medications: challenges and opportunities. Front Med (Lausanne). 2025;12:1584799. 10.3389/fmed.2025.1584799. 10.3389/fmed.2025.1584799 PMC12277326 40692948

[CR19] 19. Patton MQ. Qualitative research & evaluation methods. 4 ed. Thousand Oaks, California: SAGE Publications, Inc.; 2015.

[CR20] 20. Braun V, Clarke V. Thematic analysis - a practical guide. London, United Kingdom: SAGE; 2022.

[CR21] 21. Garvey WT, Mahle CD, Bell T, Kushner RF. Healthcare Professionals’ perceptions and management of obesity & knowledge of glucagon, GLP-1, GIP Receptor agonists, and dual agonists. Obes Sci Pract. 2024;10(3):e756. 10.1002/osp4.756. 10.1002/osp4.756 PMC11069397 38708040

[CR22] 22. Kurevija T, Šojat D, Bilić-Ćurčić I, Canecki-Varžić S, Trtica-Majnarić L. Barriers in prescribing antidiabetic medications with cardiovascular benefits: practice, experience, and attitudes of GPs in Croatia. BMC Prim Care. 2025;26(1):143. 10.1186/s12875-025-02837-7. 10.1186/s12875-025-02837-7 PMC12046718 40316896

[CR23] 23. Fredriksson M. Universal health coverage and equal access in Sweden: a century-long perspective on macro-level policy. Int J Equity Health. 2024;23(1):111. 10.1186/s12939-024-02193-5. 10.1186/s12939-024-02193-5 PMC11134649 38807180

[CR24] 24. Heitmann BL. The impact of novel medications for obesity on weight stigma and societal attitudes: a narrative review. Curr Obes Rep. 2025;14(1):18. 10.1007/s13679-025-00611-5. 10.1007/s13679-025-00611-5 PMC11799028 39907856

[CR25] 25. Post SM, Persky S. The effect of GLP-1 receptor agonist use on negative evaluations of women with higher and lower body weight. Int J Obes (Lond). 2024;48(7):1019–1026. 10.1038/s41366-024-01516-4. 10.1038/s41366-024-01516-4 PMC12439114 38561488

[CR26] 26. Tomiyama AJ. Behavioral medicine in the GLP-1 era. Ann Behav Med. 2025;59(1). 10.1093/abm/kaae069. 10.1093/abm/kaae069 PMC11783292 39657161

[CR27] 27. Dixon JB, Abdul Ghani R, Sbraccia P. Perceptions of obesity among healthcare professionals and policy makers in 2023: results of the Global OPEN Survey. Obes Sci Pract. 2025;11(1):e70033. 10.1002/osp4.70033. 10.1002/osp4.70033 PMC11707619 39781548

[CR28] 28. Cohen RV, Prager G, le Roux CW, Lingvay I, Salminen P. International federation for the surgery of obesity statement on metabolic bariatric surgery after pharmacotherapy-induced weight loss in clinical obesity. Lancet Diabetes Endocrinol. 2025;13(9):733–736. 10.1016/S2213-8587(25)00198-6. 10.1016/S2213-8587(25)00198-6 40712620

[CR29] 29. Maan S, Sohail AH, Sulaiman SA, Mansoor L, Cohen EM, Adekolu AA, et al. Metabolic and bariatric surgery versus glucagon-like peptide-1 receptor agonist therapy: a comparison of cardiovascular outcomes in patients with obesity. Am J Surg. 2025;242:116242. 10.1016/j.amjsurg.2025.116242. 10.1016/j.amjsurg.2025.116242 39965476

[CR30] 30. Dicker D, Sagy YW, Ramot N, Battat E, Greenland P, Arbel R, et al. Bariatric metabolic surgery vs glucagon-like peptide-1 receptor agonists and mortality. JAMA Netw Open. 2024;7(6):e2415392. 10.1001/jamanetworkopen.2024.15392. 10.1001/jamanetworkopen.2024.15392 PMC11161844 38848064

